# New Insight into Natural Extracellular Matrix: Genipin Cross-Linked Adipose-Derived Stem Cell Extracellular Matrix Gel for Tissue Engineering

**DOI:** 10.3390/ijms21144864

**Published:** 2020-07-09

**Authors:** Batzaya Nyambat, Yankuba B. Manga, Chih-Hwa Chen, Uuganbayar Gankhuyag, Andi Pratomo WP, Mantosh Kumar Satapathy, Er-Yuan Chuang

**Affiliations:** 1 Graduate Institute of Biomedical Materials and Tissue Engineering, School of Biomedical Engineering, College of Biomedical Engineering, Taipei Medical University, Taipei 11031, Taiwan; batzaya.nyambat@gmail.com (B.N.); yanks2020a@tmu.edu.tw (Y.B.M.); uuganbayarrrr@gmail.com (U.G.); mantoshbiotech@gmail.com (M.K.S.); 2 International Master/Ph.D. Program in Medicine, School of Medicine, College of Medicine, Taipei Medical University, Taipei 11031, Taiwan; pratomoandiwp@gmail.com; 3 Research Center of Biomedical Device, Taipei Medical University, Taipei 11031, Taiwan; 4 Department of Orthopedics, Taipei Medical University–Shuang Ho Hospital, 291 Zhongzheng Rd., Zhonghe District, New Taipei City 11031, Taiwan; 5 Cell Physiology and Molecular Image Research Center, Taipei Medical University–Wan Fang Hospital, 111, Sec. 3, Xinglong 11 Road, Wenshan District, Taipei 116, Taiwan

**Keywords:** cell-derived, extracellular matrix gel, genipin, cross-linked, tissue engineering

## Abstract

The cell-derived extracellular matrix (ECM) is associated with a lower risk of pathogen transfer, and it possesses an ideal niche with growth factors and complex fibrillar proteins for cell attachment and growth. However, the cell-derived ECM is found to have poor biomechanical properties, and processing of cell-derived ECM into gels is scarcely studied. The gel provides platforms for three-dimensional cell culture, as well as injectable biomaterials, which could be delivered via a minimally invasive procedure. Thus, in this study, an adipose-derived stem cell (ADSC)-derived ECM gel was developed and cross-linked by genipin to address the aforementioned issue. The genipin cross-linked ADSC ECM gel was fabricated via several steps, including rabbit ADSC culture, cell sheets, decellularization, freeze–thawing, enzymatic digestion, neutralization of pH, and cross-linking. The physicochemical characteristics and cytocompatibility of the gel were evaluated. The results demonstrated that the genipin cross-linking could significantly enhance the mechanical properties of the ADSC ECM gel. Furthermore, the ADSC ECM was found to contain collagen, fibronectin, biglycan, and transforming growth factor (TGF)-β1, which could substantially maintain ADSC, skin, and ligament fibroblast cell proliferation. This cell-derived natural material could be suitable for future regenerative medicine and tissue engineering application.

## 1. Introduction

Gel, a material made up of a three-dimensional cross-linked polymer chain or colloidal network within the fluid [1], is continuously attracting research interest in tissue engineering, as well as regenerative medicine. There is a clear transformation from homogeneous into more complex gels in both naturally derived and synthetic gels [2,3]. In natural gels, homogeneous extracellular matrix (ECM) constituents such as collagens, fibrin, and laminin-111 are broadly used. Nonetheless, the complexity of ECM composition and structure is not preserved. Thus, decellularized tissue-derived ECM gels, which are composed of complex proteins, proteoglycans, and growth factors, are extensively utilized [3].

Currently, tissue-derived ECM is processed into various forms, such as powder, patch, sponge, and gel [4]. Of these different forms, the gel has the potential to be created into a three-dimensional (3D) scaffold, while it can also be directly injected to the injury site for cell culture and tissue engineering applications [5]. These tissue-derived ECM gels are fabricated from different tissues such as porcine small intestine submucosa (SIS) [6], porcine urinary bladder [7], liver [8], brain [9], meniscus [10], human pancreas [11], tendon [12], and adipose tissue [13]. The development of ECM gel is associated with ECM solubilization by enzymatic (pepsin) digestion. Pepsin digests ECM into several incompletely digested proteins, proteoglycans, growth factors, and matricellular proteins, including collagens, elastin, laminin, fibronectin, hyaluronan, heparan, basic fibroblast growth factor (bFGF), vascular endothelial growth factor (VEGF), insulin-like growth factor (IGF), transforming growth factor (TGF)-β, tenascin, osteopontin, and thrombospondin [5,7,10,13]. Among them, a major component of ECM is collagen, which is involved in processes of cell growth, proliferation, migration, and differentiation through inducing cell signalization. In addition, collagen has attractive characteristics, including low inflammatory and cytotoxic responses, as well as high biodegradability [14,15]. It is polymerized by the self-assembly process of the fibrillar structure to generate a gel at body temperature (37 °C) and neutral pH (7.4). The collagen fiber cross-linking is promoted by hydrophobic and electrostatic interactions [16,17,18,19]. This temperature- and pH-dependent sol–gel state of collagen in ECM allows an injectable ECM gel [17]. Furthermore, the delivery method of ECM gel with minimum invasive and uncomplicated procedures is beneficial for both patients and clinicians [19]. However, tissue-derived ECM has the risk of pathogen transfer and uncontrollable variability emerging from the age and the healthiness of individual donors [20,21].

Therefore, cell-derived ECMs, possessing an ideal niche with growth factors and complex fibrillar proteins for cell attachment and growth without the risk of pathogen transfer, are utilized as an alternative source of ECM in tissue engineering [20,21,22]. Moreover, cell-derived ECMs can avoid uncontrollable variability by selecting necessary cell sources, culture media, and culture systems [20,23,24]. Presently, cell-derived ECMs are obtained from fibroblast [25], chondrocytes, bone marrow-derived mesenchymal stem cells (BMSCs) [21], and adipose-derived stem cells (ADSCs) [26] using different decellularization methods. Among them, ADSCs have easy accessibility, a high obtained cell percentage [27], and increased collagen production [28]. In addition, ADSCs and ADSC ECMs [29] showed promising results in wound healing [30], ischemic heart disease [31], tendon [32], anterior cruciate ligament (ACL) healing [33], bone regeneration [34], and anti-cancer study [35] through their differentiation, paracrine, pro-angiogenic, and immunomodulatory properties. Nonetheless, the processing of cell-derived ECM into gels is scarcely studied [36]. Theoretically, the formulation of a cultured cell-derived ECM gel as an ideal three-dimensional material could be an appropriate microenvironment for cell attachment and growth [25].

Furthermore, cultured cell-derived ECMs have concerns regarding poor mechanical properties and the rapid degradation of active therapeutic substances, which are major disadvantages for tissue healing [24,37]. Controlling the biodegradability rates and preserving therapeutics are extremely crucial. Currently, most biomaterials’ mechanical properties are increased using chemical cross-linking agents, including glutaraldehyde, 1-ethyl-3-carbodiimide, and formaldehyde. Several of them have lower cytocompatibility in comparison with the natural cross-linking agent [38,39]. Hence, the genipin naturally derived cross-linking agent is utilized extensively due to its minimal cytotoxicity, as well as high cross-linking degree [40,41,42,43,44]. Additionally, genipin cross-linked hydrogels (gels) achieved tunable biomechanical properties along with functional tissue regeneration and less inflammatory reaction based on the ECM free amino group conjugation [45]. Therefore, this study aimed to develop an ADSC ECM gel and reinforce its biomechanical properties using genipin cross-linking for tissue engineering.

## 2. Results

### 2.1. Materials Characterization

#### 2.1.1. Physicochemical Analysis

**Figure 1:**
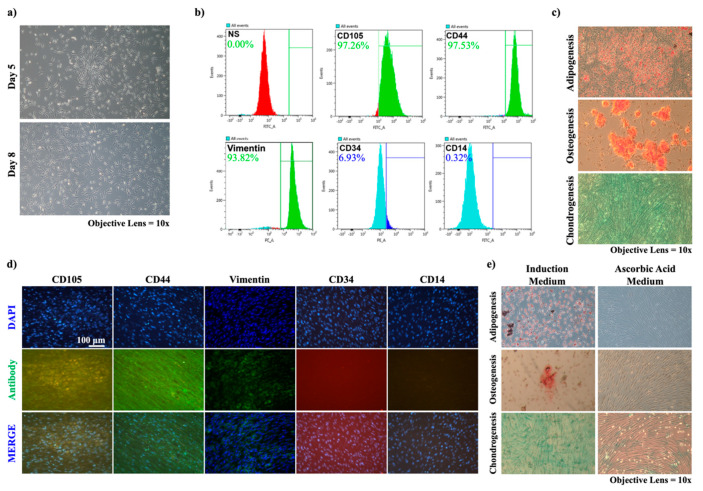
Cell characterization: (**a**) morphology; (**b**) flow cytometry analysis of harvested cells; (**c**) differentiation analysis of adipose-derived stem cells (ADSCs) with oil red O (lipid vacuoles), Alizarin red (calcium deposition), and Alcian blue staining (proteoglycan); (**d**) Fluorescence microscopy observation for ADSC sheets after 10 days of culture (scale bar = 100 µm); (**e**) Light microscopy visualization of the undifferentiated state of ADSCs in cell sheets cultured with ascorbic acid, along with differentiation of ADSCs in cell sheets cultured with induction medium into adipocytes, osteocytes, and chondrocytes.

The cultured cell-derived ECM gel was developed from rabbit subcutaneous fat tissue-derived ADSCs, as described in Section 4. The microscopic observation indicated that the ADSCs had a spindle-shaped morphology and formed colonies after five days of the primary culture. The colony size and cells in the colonies were uniform, and ADSCs reached 80–100% confluence after eight days of culture (Figure 1a). Surface markers and the intracellular vimentin of harvested cells were detected using flow cytometry analysis. As depicted in Figure 1b, most of the cultured cells expressed cluster of differentiation 105 (CD105) and CD44 surface markers, as well as intracellular vimentin. In contrast, cells had low expression of CD34 and CD14 (hematopoietic cell marker) surface markers. Further, the adipogenic, osteogenic, and chondrogenic differentiation of the harvested ADSCs were assessed using different induction medium. As shown in Figure 1c, ADSCs could differentiate into adipocytes, osteocytes, and chondrocytes after 21 days of culture.

Furthermore, ADSCs sheets were successfully produced by culturing the cells with 50 µg/mL ascorbic acid for 10 days. The fluorescent microscopic observation indicated that surface markers and the intracellular vimentin of ADSCs in cell sheets such as CD105, CD44, and vimentin were still positive after 10 days of culture (Figure 1d). The undifferentiated state of ADSCs in cell sheets was detected using oil red O, Alizarin red, and Alcian blue staining following 10 days of culture with ascorbic acid or induction medium. Figure 1e shows the results of oil red O, Alizarin red, and Alcian blue staining for adipogenic, osteogenic, and chondrogenic differentiation, respectively. In ADSCs treated with induction medium, lipid vacuoles, calcium nodules, and proteoglycan were formed after 10 days, while ADSCs cultured with ascorbic acid showed no differentiation.

**Figure 2:**
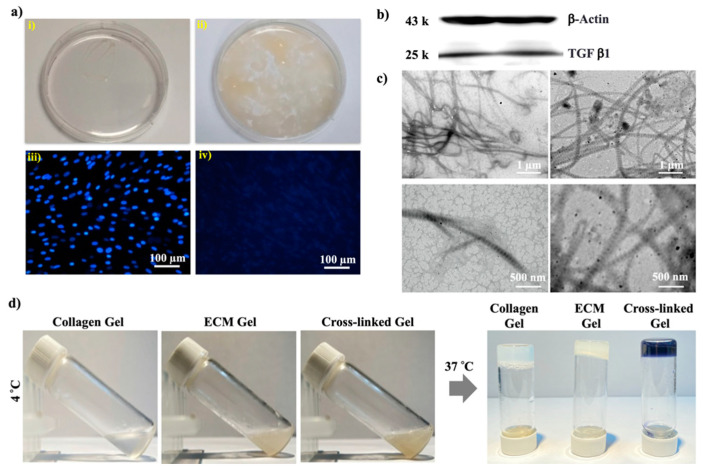
Cell sheet characterization: (**a**) (i) single ADSC sheet, (ii) ASDC sheet collection, (iii) ADSC sheet with typical round-shaped nuclei stained with 4′6-diamidino−2-phenylindole (DAPI), and (iv) decellularized ADSC sheets with irregularly shaped nuclei and weak DAPI staining; (**b**) transforming growth factor (TGF)-β1 in the extracellular matrix (ECM) by Western blotting analysis; (**c**) TEM analysis of collagen and ECM gel; (**d**) gross observation immediately after adding genipin (4 °C) and gel formation at 37 °C.

Subsequently, the ADSCs sheets were separated from the culture dishes effortlessly by washing with phosphate-buffered saline (PBS) and a cell scraper (Figure 2a(i,ii)). ADSCs were uniformly distributed throughout the cell sheets by fluorescent microscopic examination. Decellularization efficiency was validated by 4′6-diamidino−2-phenylindole (DAPI) staining of the cell nuclei in the cell sheet. Cell nuclei in the ADSC sheet showed a typical round shape based on DAPI staining (Figure 2a(iii)). In contrast, cell nuclei in the decellularized ADSC sheet revealed an irregular shape with weak staining of DAPI (Figure 2a(iv)).

Moreover, the Western blotting analysis revealed that the ADSC-derived ECM still contained TGF-β1 after decellularization (Figure 2b). TEM was employed to observe the “D” periodicity in the collagen gel and developed ADSC ECM gel. As depicted in (Figure 2c), collagen fibrils were formed in both collagen and ADSC ECM gels. Figure 2c (left) shows the collagen gel (control), and Figure 2c (right), shows the ADSC ECM gel. As demonstrated in (Figure 2d), gross observation of the sol–gel formation revealed that collagen, the ADSC ECM, and the genipin cross-linked ADSCs ECM could be gelated from 4 °C to 37 °C. Collagen and the ADSC ECM gel had an opaque appearance, whereas the genipin cross-linked ADSC ECM gel had a dark-blue color.

To examine the chemical structure of the ADSC ECM, mass spectrometry analysis was performed. Mass spectrometry analysis showed that collagen type-I, biglycan, fibronectin, decorin, and vitronectin were retained in the ECM along with other proteins (Table 1).

#### 2.1.2. SEM, Fourier-Transform Infrared (FTIR), and Ninhydrin Assay

**Figure 3:**
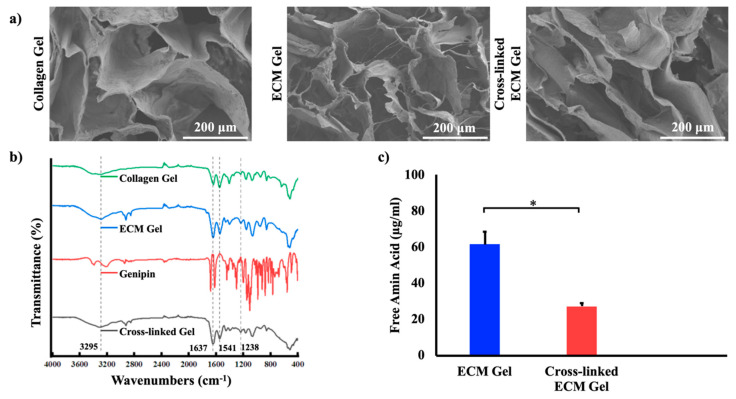
(**a**) Representative SEM images of the collagen, ECM, and cross-linked ECM gels; (**b**) Fourier-transform infrared (FTIR) analysis; (**c**) free amino acid determination using ninhydrin assay (*n* = 3), showing a significant decrease of free amino acid amount in cross-linked ECM gel (* *p* < 0.05).

The ultra-structure of the ADSC ECM and genipin cross-linked ADSC ECM gels was observed through SEM, which indicated a highly porous microstructure in all groups (Figure 3a). FTIR was employed to examine the molecular structure of the genipin cross-linked ADSC ECM gel composite. The typical bands of amide I, II, and III for the ADSC ECM gel were observed at the respective bands of 1637 cm^−1^, 1541 cm^−1^, and 1238 cm^−1^, caused by stretching vibrations of the carbonyl groups (C=O bond), a combination of C–N stretching and amide N–H in-plane bending vibrations, and CH_2_ wagging vibration. The N–H and O–H stretching vibrations at 3295 cm^−1^ peak, which could be due to the overlapping bands. Compared with the ADSC ECM gel, the cross-linked ADSC ECM gel spectrum decreased in the characteristic peak of amine (–NH_2_) stretch at 1541 cm^−1^ (Figure 3b). Moreover, the ninhydrin assay showed that, after genipin cross-linking, the free amino acids of the ADSC ECM gel were significantly reduced (Figure 3c), and the cross-linking degree was 55.9%.

#### 2.1.3. Rheological and Degradation Analyses

**Figure 4:**
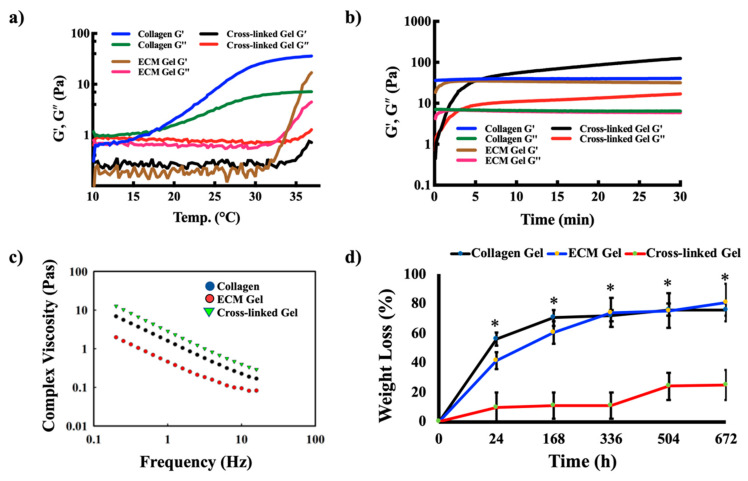
Rheological and degradation analysis of collagen, ADSC ECM, and cross-linked ADSC ECM gels: (**a**,**b**) gelation kinetics by temperature and time sweep; (**c**) viscosity vs. frequency plots; (**d**) degradation in simulated body fluid (SBF) (*n* = 3). The cross-linked gel had significantly lower the degradation rate than the collagen and ECM gels (* *p* < 0.05).

The flow behavior of the collagen, ADSC ECM, cross-linked ADSC ECM gels was figured out using rheological measurements. The findings indicated that both the storage modulus (G′) and the loss modulus (G″) increased with the temperature rise from 10 °C to 37 °C in collagen and ADSC ECM gel group (Figure 4a). In contrast, the cross-linked ADSC ECM gel had a time-dependent increase at 37 °C (Figure 4b). At the initial gelation, G′ was higher than G″ (G′ > G″) indicating that the gels had a solid-like structure in all collagen, ADSC ECM, and cross-linked ADSC ECM gels. In Figure 4c, the complex viscosity (η*) of the cross-linked ECM gel was highest in comparison with the collagen and ADSC ECM gels. The degradation tendency of the developed gel was measured for the mass loss of collagen, ADSC ECM, and cross-linked ADSC ECM gels in simulated body fluid (SBF). During the degradation analysis (Figure 4d), the cross-linked ADSC ECM gel had significantly lower the degradation rate compared with the collagen and ADSC ECM gels with *p* < 0.05 at all time points.

### 2.2. In Vitro Analysis

**Figure 5:**
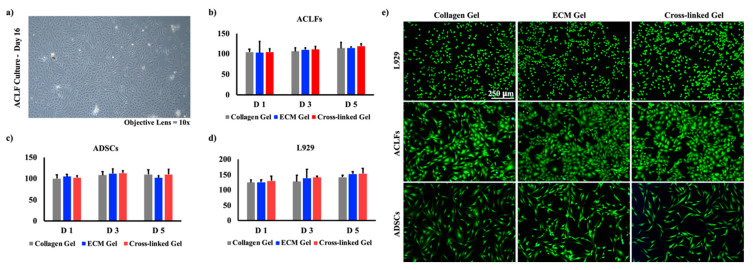
(**a**) Primary anterior cruciate ligament (ACL) fibroblast colony formation; (**b**–**e**) ACL fibroblast, ADSC, and L929 cell 3-(4,5-dimethylthiazol-2-yl)-2,5-diphenyltetrazolium bromide (MTT) and Calcein acetoxymethyl (AM) cell viability assay, respectively (*n* = 3). There were no significant differences in cell morphology and viability among all gels groups (* *p* > 0.05).

To assess the cytotoxic efficacy of the genipin cross-linked ADSC ECM gel, 3-(4,5-dimethylthiazol-2-yl)-2,5-diphenyltetrazolium bromide (MTT) and Calcein acetoxymethyl (AM) assays were employed on L929 mouse skin fibroblasts and rabbit primary anterior cruciate ligament fibroblasts (ACLFs). Primary ACLFs were harvested from a rabbit ACL tissue, forming colonies on day 16 (Figure 5a). Furthermore, the MTT assay was performed to analyze cytocompatibility.

As mentioned in Section 4, collagen, the ADSC ECM, and cross-linked ADSC ECM gels were incubated in the different cell culture media (DMEM/high glucose [HG] or DMEM/low glucose [LG]) for one, three, and five days, and then the medium was extracted. Furthermore, primary ACL fibroblasts, ADSCs, and L929 fibroblast cells were seeded individually and incubated until reaching confluency, which were further treated using each extracted medium for 24 h. The MTT assay showed that the viability of ACL fibroblasts (Figure 5b), ADSCs (Figure 5c), and L929 cells (Figure 5d) was maintained in all groups. To confirm the cytocompatibility of the ADSC ECM and cross-linked ADSC ECM gels, Calcein AM analysis (Figure 5e) was carried out with the L929 cells, ACLFs, and ADSCs treated with extraction medium (5 days) using collagen, ADSC ECM, and cross-linked ADSC ECM gels for 24 h.

Finally, fluorescent microscopic observation revealed that L929 cells, ACLFs, and ADSCs had high viability in the presence of all extraction media using collagen, ADSC ECM, and cross-linked ADSC ECM gels after one day of culture (Figure 5e).

## 3. Discussion

There is an increasing need for pathogen-free natural scaffolds with satisfactory physicochemical properties in regenerative medicine, as well as tissue engineering. Cell-derived ECM scaffolds have excellent biophysicochemical properties that closely mimic the native ECM microenvironment. They are produced in vitro from various cell types, as a consequence of avoiding pathogen transfer. However, the application of cell-derived ECMs is limited due to their inferior mechanical properties. In the present study, a novel ECM gel from rabbit ADSCs was fabricated, and its mechanical properties were reinforced by genipin cross-linking.

**Scheme 1:**
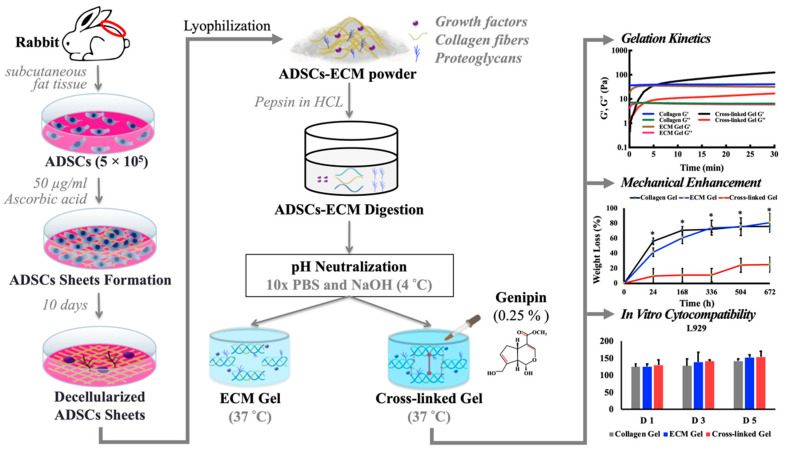
Material preparation and experimental overview of the study. The asterisk (*) indicates the statistically significant difference between collagen, ADSC ECM, and cross-linked ADSCs ECM gels (* *p* < 0.05).

The physicochemical properties of the genipin cross-linked ADSC ECM gel was assessed, and the cytocompatibility for the mouse skin fibroblasts (L929), rabbit primary ACLFs and ADSCs was evaluated. The ADSC ECM gel was produced via sequential processes such as ADSC isolation, expansion, ADSC sheet preparation, decellularization of the cell sheet, lyophilization of the ECM, enzymatic digestion, neutralization of the ECM solution, and genipin cross-linking (Scheme 1). Initially, the surface markers, intracellular vimentin, and multilineage differentiation potential of the harvested cells were validated. It is reported that the ADSCs express CD105, CD44, and vimentin, and they possess the ability to differentiate into multiple lineages [46,47]. In our findings, the harvested cells were positive for CD105 and CD44 surface markers, as well as intracellular vimentin, indicating that the harvested cells from rabbit subcutaneous fat tissue were ADSCs, consistent with the previous studies [46,47].

Moreover, stem cells have the ability to differentiate into multilineages while maintaining the undifferentiated state [48]. Therefore, the adipogenic, osteogenic, and chondrogenic differentiation potential of the harvested ADSCs was assessed using oil red O, Alizarin red, and Alcian blue staining. The results showed that ADSCs could differentiate into adipocytes, osteocytes, and chondrocytes. Furthermore, ADSC sheets were successfully produced by culturing the cells with ascorbic acid for 10 days. The ascorbic acid was found to be useful for cell sheet formation by stimulating telomerase activity in stem cells to increase ECM production [49]. Then, the undifferentiated state of ADSCs in the cell sheet was confirmed by the positive expression of CD105 and CD44 surface markers, as well as intracellular vimentin. Some studies revealed that ADSCs could differentiate into adipogenic [50], osteogenic, and chondrogenic lineages [51] after adding ascorbic acid [52] and long-term culturing [53,54], which has the risk of unwanted tissue formation. Hence, the undifferentiated state of ADSCs in cell sheets was confirmed using oil red O, Alizarin red, and Alcian blue staining following 10 days of culture with ascorbic acid or induction medium. According to the results, in the ADSCs treated with induction medium, lipid vacuoles, calcium nodules, and proteoglycan were formed after 10 days, while, in the ADSCs cultured with ascorbic acid, no differentiation was observed. Thus, this implies the ADSCs in cell sheets maintained an undifferentiated state due to the concentration of the ascorbic acid [55] and the use of FGF-2 [56] in the ADSC culture medium.

Subsequent decellularization of ADSC sheets showed that the ECM contained collagen type I, biglycan, fibronectin, decorin, vitronectin, and TGF-β1 using mass spectrometry and Western blotting analysis. These findings indicate that the ADSC ECM gel contained numerous essential ECM proteins, which dominate the tissue and organs [57,58]. Recently, a study showed that the ECM and conditioned medium of a 3D ADSC culture contains a high amount of collagen type I [29,59], which is consistent with our study finding. In addition, ECM scaffolds containing collagen and glycosaminoglycans (GAGs) are extensively involved in soft tissue engineering, including skin, heart, tendon, and ligament, and they showed superior results [60,61,62,63]. Furthermore, collagen type I is a significant component of ECM and collagen, whereby fibrils are formed from the spontaneous aggregation of tropocollagen molecules into ordered arrays. This order generates a prominent 60–70-nm transverse “D” periodicity [64]. In the present study, TEM images indicated the “D” periodicity in both the collagen gel and the developed ADSC ECM gel.

Moreover, collagen type I in ECM is ideally polymerized at body temperature (37 °C) and neutral pH (7.4) [17,19]. Based on this phenomenon, the ADSC ECM and genipin cross-linked ADSC ECM formed gels from 4 °C to 37 °C. A dark-blue color was produced from the genipin cross-linked ADSC ECM gel, which appeared due to a reaction between amine groups of the ECM and genipin [43].

Following the development of ADSC ECM and genipin cross-linked ADSC ECM gels, SEM visualized a highly porous microstructure. Studies reported that larger pore sizes (100–500 µm) increase cell migration and metabolic activity, along with angiogenesis, as well as transportation of sufficient nutrients [60,65]. Thus, our developed, porous gel might be suitable for cell migration and growth. The FTIR spectrum showed the typical bands of amide I, II, and III for the cross-linked ADSC ECM gel, and the presence of amide III relating to C-N stretching and NH reveals the triple helical structure of collagen is preserved [66,67]. Moreover, the spectrum of the cross-linked ADSC ECM gel showed a decrease in the characteristic peak of amine (–NH_2_) stretch at 1541 cm^−1^, and this result was consistent with genipin cross-linked tissue-derived ECM [68]. Furthermore, genipin cross-linking increased the stability of a human umbilical cord-derived ECM gel in a dose-dependent manner based on the conjugation of free amino groups of the ECM [45]. In this study, the ninhydrin assay showed that the genipin cross-linking significantly decreased the free amino acids of the ADSC ECM gel, as a result of the 55.9% cross-linking degree. It was reported that a lower amount of free amino acids after cross-linking indicates an effective cross-linking of biomaterials [69]. This finding is in accordance with a previous study [45].

The flow behavior of the collagen, ADSC ECM, and cross-linked ADSC ECM gels indicated that both the storage modulus (G′) and the loss modulus (G″) increased with temperature from 10 °C to 37 °C in the collagen and ADSC ECM gel groups. In contrast, the cross-linked ADSC ECM gel had a time-dependent increase at 37 °C. Furthermore, the complex viscosity (η*) of the cross-linked ECM gel was highest in comparison with the collagen and ADSC ECM gels. Implying that, the cross-linked ADSC ECM gel was stiffer than the collagen and ADSC ECM gels. The degradation analysis revealed that the cross-linked ADSC ECM gel had significantly lower degradation compared with the collagen and ADSC ECM gels. These results suggest that genipin cross-linking could enhance ECM mechanical properties. The increased stiffness and slow degradation of the cross-linked ADSC ECM gel were attributed to the dense cyclic intermolecular cross-linking between collagen and genipin with additional hydrogen bonding [70].

In view of the potential applications of the genipin cross-linked ADSC ECM gel for tissue engineering, rabbit primary ACL fibroblasts, ADSCs, and the standard cell toxicity cell line L929 were used to evaluate cytocompatibility. In the indirect MTT and Calcein AM assays, both the ADSC ECM and the cross-linked ADSC ECM gels maintained cell morphology and viability. Thus, their cytocompatibility was likely due to the minimal cytotoxicity of genipin, which is about 10,000 times more cytocompatible than glutaraldehyde [71].

We presume that our developed gel containing TGF-β1 along with other essential ECM proteins, with suitable biodegradation and mechanical properties, might lead to better healing for future applications.

## 4. Materials and Methods

### 4.1. Materials

The chemicals used in genipin cross-linked ADSC-ECM gel fabrication were of standard purity levels. Collagenase type I (0.075%), and l-ascorbic acid 2-phosphate sesquimagnesium salt hydrate were from Sigma-Aldrich, Taipei City, Taiwan. Furthermore, the cell culture media, reagents, antibodies, and related buffers were purchased from main suppliers and distributors of Thermo Fisher Scientific (Taipei City, Taiwan), GeneTex (Hsinchu City, Taiwan), Biolegend (Taipei City, Taiwan), Simply Biologics (Miaoli County, Taiwan), Challenge Bioproduct (Touliu City, Yunlin, Taiwan), Biological Industries (New Taipei City, Taiwan), Santa Cruz Biotechnology (Dallas, TX, USA), and Corning (Taipei City, Taiwan). Most chemicals and reagents used in this research were of analytical grade obtained from Sigma-Aldrich.

### 4.2. ADSC Cell Sheet and ECM Isolation and Preparation

ADSCs isolation was performed similarly to our previous publication [26]. Back subcutaneous fat tissues from New Zealand male rabbits were harvested after euthanasia with carbon dioxide (CO_2_) inhalation. After removing blood vessels and outer fibrous membranes, fat tissue was cut and digested with collagenase type I. Cells were cultured in DMEM/low glucose (LG) containing 10% fetal bovine serum (FBS), 50 µg/mL penicillin, 50 µg/mL streptomycin, 100 µg/mL amphotericin, and 20 µg/mL FGF-2 in a humidified atmosphere of 5% CO_2_ and 95% air at 37 °C. ADSCs were subcultured around 80% confluence [72], and passage three was used in all experiments. The differentiation potential of ADSCs was assessed by culturing the cells in adipogenic, osteogenic, and chondrogenic differentiation medium for 21 days after confirming surface markers through flowcytometry. Then, the cells were stained using oil red O (lipid droplet), Alizarin red (calcium nodule), and Alcian blue (proteoglycan) according to standard protocol. The ADSCs morphology and differentiation were observed and recorded by ZEISS microscopy (Jena, Germany).

Similarly to the previous publication [37], the ADSC ECM was prepared by culturing ADSCs (5 × 10^5^ cells/well) with 50 µg/mL ascorbic acid (l-ascorbic acid 2-phosphate sesquimagnesium salt hydrate) and 20 µg/mL FGF-2 in a 10-cm dish for 10 days to form cell sheets (under the same conditions as previously mentioned) [49,73,74]. The culture medium comprising of ascorbic acid was changed every three days. After 10 days of culture, cell sheets were separated from the dishes by PBS washing and a cell scraper. Furthermore, the ADSC sheets were decellularized similarly to our previous publication [26]. The decellularization was visualized using 300 nM 4′6-diamidino-2-phenylindole staining under fluorescence microscopy (Olympus IX71, Tokyo, Japan). Finally, the ADSC ECM powder was made after 24 h of vacuum drying and grinding.

### 4.3. ADSC and ADSC Cell Sheet Identification by Flowcytometry and Immunofluorescent (IF) Staining

The surface markers of the harvested cells were detected using a spectral flow cytometry cell analyzer (Sony SA 3800; San Jose, CA, USA), which accounts for 10^4^ cells/sample. The cells (1 × 10^6^) at passage three were incubated with the subsequent antibodies: fluorescein isothiocyanate (FITC)-conjugated mouse monoclonal antibodies against CD105, anti-rabbit CD44 rat monoclonal primary antibody and anti-rat rabbit polyclonal secondary antibody, phycoerythrin (PE)-conjugated mouse monoclonal antibody against rabbit vimentin, and PE-conjugated rat and FITC-conjugated mouse monoclonal antibody against CD34 and CD14 antibodies in PBS containing 1% bovine serum albumin (BSA). The unstained cells were used as controls. Then, to identify surface markers and intracellular vimentin of ADSCs in cell sheets, immunofluorescent (IF) staining was performed after 10 days of culture with ascorbic acid. Briefly, the cell sheets were fixed in 4% paraformaldehyde for 10 min at room temperature. Prior to CD marker visualization by fluorescent microscopy, the cell sheets were washed with PBS and incubated with the previously mentioned antibodies. Finally, to demonstrate the undifferentiated state of ADSCs in cell sheets, oil red O (lipid droplet), Alizarin red (calcium nodule), and Alcian blue (proteoglycan) staining was conducted after 10 days of culture with ascorbic acid. ADSCs cultured in adipogenic, osteogenic, and chondrogenic differentiation medium for 10 days served as the positive control group.

### 4.4. ADSC ECM Mass Spectrometry Analysis

Mass spectrometry analysis was accomplished to identify the chemical composition of the ADSC ECM. The ECM solution was prepared by sonicating in 0.1% acetic acid solution to measure the protein concentration by Bradford assay. Then, solutions containing 10 µg of ECM protein were reduced to a 10-µL mixture of 2 mM dithioerythritol, 8 M urea, and 25 mM ammonium bicarbonate, heating at 37 °C for 1 h. The free cysteine residues were prevented from disulfide bond reformation by labeling with 10 µL of 20 mM iodoacetamide for 1 h at room temperature in the dark. Prior to adding trypsin (50:1, *w*/*w*), samples were diluted to final concentrations of 1 M urea, followed by quenching the reaction with 10 µL of 1% formic acid to deactivate any unreacted reagents. Peptides of the ECM solution were purified with a mixture of 50% acetonitrile and 0.1% formic acid passed through a C18 Zip-Tip. Finally, samples were eluted with a mixture of 50% acetonitrile and 0.1% formic acid and dried in a speed vacuum device. Spectra of the samples were recorded using electrospray ionization (ESI) quadrupole time-of-flight (QUAD-TOF) MS/MS analysis (Waters SYNAPT G2, Milford, MA, USA). To validate the protein identifications and MS/MS-based peptide, protein samples were analyzed by using Mascot (Matrix Science, London, UK).

### 4.5. Western Blotting Assay

The Western blotting analysis was carried out to detect TGF-β1 in the ADSC ECM. Briefly, ADSC ECM powders were homogeneously sonicated in PBS to attain the ADSC ECM solution. Proteins were separated from the gel with 8% polyacrylamide by electrophoresis to a polyvinylidene difluoride membrane. Nonspecific binding was blocked by soaking membranes in Tris-buffered saline (TBS), 5% powdered milk, and 0.1% Tween 20 at room temperature for 1 h. Later, the membranes were incubated with anti-β-actin primary antibody (1:1000) and TGF beta antibody (TB21; mouse monoclonal antibody (1:1000)) overnight. Furthermore, after washing with the mixture of TBS and Tween 20 and the membrane was incubated with horseradish peroxidase (HRP)-conjugated secondary antibody. Detection was performed using the bispectrum multispectral imaging system (Analytik Jena US LLC).

### 4.6. Genipin Cross-Linked ADSC ECM Gel Preparation

Firstly, 45 mg of ADSC ECM was digested using pepsin (~2000 U/mg) in 1 mL of 0.01 M hydrochloric acid at room temperature for 3 h. Later, the ECM solution was neutralized after adding 10 × PBS and 0.1 N sodium hydroxide on ice. The genipin cross-linked ADSC ECM gel was achieved by mixing 0.25% (*w*/*v*) genipin with the ECM solution. Finally, the ADSC ECM and cross-linked ADSC ECM gels were formed at 37 °C under a CO_2_ incubator after transferring the ECM solution with and without genipin. Commercial collagen (rat tail-derived collagen type I) was prepared according to the manufacturer’s instruction and served as the control.

### 4.7. Transmission Electron Microscopy (TEM) and Scanning Electron Microscopy (SEM)

For TEM, to analyze collagen fiber formation, the collagen and ADSC ECM gels were transferred onto Formvar-coated copper grids. After lyophilization, the gels were stained using 1% (*w*/*v*) negative stain and the morphology was observed using TEM (HT7700, Hitachi, Japan). The ultrastructure of the collagen, ADSC ECM, and genipin cross-linked ADSC ECM gels were examined using SEM (Hitachi SU3500).

### 4.8. Fourier-Transform Infrared (FTIR)

The FTIR spectroscopy (Thermo Fisher Scientific, ST, USA) assay was done to demonstrate the composition of the developed ECM gels. The collagen, ADSC ECM, and genipin cross-linked ADSC ECM gels were vacuum-dried and ground. Spectra of the samples were recorded.

### 4.9. Ninhydrin Assay

The cross-linking degree and free amino content of the genipin cross-linked ADSC ECM gel were analyzed using the ninhydrin assay. Dry ADSC ECM (2–3 mg) and genipin cross-linked ADSC ECM gels were mixed with 2 mL of deionized water (dH_2_O) and 1 mL of 2% ninhydrin solution. The mixture was preheated at 100 °C for 10 min and cooled at room temperature to end the reaction. Furthermore, free amino groups were determined by spectrophotometry (Multiscan FC, Thermo Scientific) at 570 nm. Different concentrations of glycine were prepared for the standard curve, and the degree of cross-linking was determined using Equation (1) [75].

Degree of cross-linking (%) = [(NH_2_)_nc_ − (NH_2_)_c_/(NH_2_)_nc_ ] × 100 (1)

where (NH_2_)_nc_ is the mole fraction of the free amino groups in the ADSC ECM gel, and (NH_2_)_c_ is the mole fraction of the free amino groups in the genipin cross-linked ADSC ECM gel.

### 4.10. Rheology and Degradation Analysis

The rheological analysis was conducted using a TA Instruments AR2000 with a 40-mm-diameter parallel plate geometry and a Peltier cell [7]. The collagen, ADSC ECM, and genipin cross-linked ADSC ECM gels were prepared and loaded into the rheometer with the Peltier cell at 10 °C. Furthermore, the temperature was adjusted to 40 °C to induce gelation. In this way, the Peltier cell ideally reached a temperature of 30 °C within 10 min and 36.9 °C at 13.4 min. The subsequent gelation with the increase in temperature of the sample was continuously detected at a fixed frequency of 0.159 Hz (1 rad/s) at a 5% strain. Then, the time sweep was analyzed at 37 °C for 30 min. Thus, when there was no further change in the elastic modulus (G′) with time, gelation was considered to be complete. The gel’s final linear viscoelastic properties were determined at a sweep frequency between 15.9 and 0.08 Hz (100–0.5 rad/s) at 37 °C and 5% strain.

The collagen, ADSC ECM, and genipin cross-linked ADSC ECM gel degradation was assessed after gel lyophilization, weighing, and further immersion into 1.5 mL of simulated body fluid (SBF) in Eppendorf tubes at 37 °C (water bath). After 24, 168, 336, 504, and 672 h, SBF was removed, and the gels were weighed following lyophilization (W_i_, initial dry sample weight, and W_t_, dry sample weight at the respective period). Finally, the degree of degradation was expressed in terms of the weight loss percentage (wt.%) of the gels according to Equation (2) [76].

W_(wt. % )_ = (W_i_ − W_t_)/W_i_ × 100 (2)

### 4.11. In Vitro Studies—Cytotoxicity Analysis

To evaluate the cytotoxic efficacy of the genipin cross-linked ADSC ECM gel, L929 mouse skin fibroblasts, rabbit primary anterior cruciate ligament fibroblasts (ACLFs), and ADSCs were utilized from New Zealand male rabbits. ADSCs were isolated and cultured under the same conditions previously mentioned.

#### 4.11.1. ACL Fibroblast Isolation

ACL fibroblasts were isolated similarly according to a previous publication [77]. ACL tissues from New Zealand male rabbits were harvested after euthanasia with CO_2_ inhalation. After removing the outer fibrous membrane, the ligament tissue was minced and digested with collagenase type I (0.2%; Sigma-Aldrich). Cells were cultured in DMEM/high glucose (HG) containing 10% FBS, 50 µg/mL penicillin, 50 µg/mL streptomycin, and 100 µg/mL amphotericin in a humidified atmosphere of 5% CO_2_ and 95% air at 37 °C. Lastly, the ACL fibroblasts were passaged when they reached between 80% and 90% confluence.

#### 4.11.2. MTT Analysis

A 3-(4,5-dimethylthiazol-2-yl)-2,5-diphenyltetrazolium bromide (MTT) assay was conducted to analyze cell metabolic activity on the collagen, ADSC ECM, and genipin cross-linked ADSC ECM gels. Briefly, 0.2 g of the gels were pre-incubated in DMEM/LG or DMEM/HG for one, three, and five days and the extracts of the medium were stored at −20 °C. Then, ADSCs, skin fibroblasts (L929), and ACLFs (5 × 10^3^) were seeded in 96-well plates. Furthermore, the cells were exposed with the extracted medium for 24 h after reaching confluence. Then, the MTT solution (5 mg/mL) was added into wells according to standard protocol, and the absorbance at 570 nm was recorded using an enzyme-linked immunosorbent assay reader (ELISA) (Thermo Scientific, Multiscan FC). The cell viability (%) normalized to the control group was calculated.

#### 4.11.3. Calcein AM Staining Analysis

To assess cytotoxicity qualitatively, a Calcein AM staining assay was performed on the ADSCs, skin fibroblasts (L929), and ACLFs using fluorescence microscopy (Leica Microsystems, CMS GmbH, Germany). Firstly, 0.2 g/mL of the collagen, ADSC ECM, and cross-linked ADSC ECM gels were incubated in DMEM/LG or DMEM/HG for five days. The ADSCs, ACLFs, and L929 cells (3 × 10^4^ cells) were seeded in 3-cm confocal dishes with complete medium for 24 h. After removing the medium, a total of 150 µL of extraction medium for the collagen, ADSC ECM, and genipin cross-linked ADSC ECM gels was added to the different cells and incubated in a 5% CO_2_ incubator at 37 °C for 24 h. Afterward, to observe cytotoxicity, the cells were stained with assay reagents (Calcein AM staining kit, Dojindo, Japan), and fluorescent signals were determined (2 µM Calcein AM at 490 ± 10 nm). Live cells were presented in green.

### 4.12. Statistical Analysis

All experiments were conducted with at least three replicates (*n = 3*). The results are reported as the average value ± standard deviation. One-way analysis of variance (ANOVA) was performed to compare multiple groups of data. Differences were considered statistically significant when *p* < 0.05. Fabrication of the genipin cross-linked ADSC ECM gel and an experimental overview of the study are schematically presented below as Scheme 1.

## 5. Conclusions

In this research, a genipin cross-linked ADSC ECM gel was successfully fabricated, which could assist cell behavior through its structural, biochemical stimulation, and biomechanical properties. Furthermore, the ADSC ECM containing collagen, fibronectin, biglycan, and TGF-β1 could maintain ADSC, skin, and ligament fibroblast cell proliferation. The mechanical properties of the genipin cross-linked ADSC ECM gel was enhanced with less degradability. Thus, this natural material could serve as a suitable biomaterial for future regenerative medicine and tissue engineering applications.

## Figures and Tables

**Table 1:** ADSC ECM components determined using LC–MS/MS analysis.

Prot_Acc ^a^	Prot_Desc ^b^	Prot_Matches ^c^	Pep_Exp_Mr ^d^
A2BDB0	Actin, cytoplasmic 2 OS = Xenopus laevis GN = actgl PE = 2 SV = 1	18	997.3894
PI 2763	Alpha-2-HS-glycoprotein OS = Bos taurus GN = AHSG PE = 1 SV = 2	4	1268.6012
C0HJG9	Annexin A2 (Fragments) OS = Mesocricetus auratus PE = 1 SV = 1	2	1541.8317
Q9GKQ6	Biglycan (Fragments) OS = Sus scrofa GN = BGN PE = 2 SV = 2	4	908.4342
PI2725	Alpha-1-antiproteinase OS = Ovis aries PE = 1 SV = 1	3	746.3114
C0HJP5	Collagen alpha-l(I) chain (Fragments) OS = Macrauchenia sp. GN = COLl A1 PE = 1 SV = 1	7	1087.4454
P12111	Collagen alpha-3(VI) chain OS = Homo sapiens GN = COL6A3 PE = 1 SV = 5	6	1740.8691
Q04857	Collagen alpha-1 (VI) chain OS = Mus musculus GN = Col6al PE = 1 SV = 1	4	1579.7097
Q28888	Decorin OS = Oryctolagus cuniculus GN = DCN PE = 2 SV = 1	7	1128.5521
Q99K41	EMILIN-l OS = Mus musculus GN = Emilinl PE = 1 SV = 1	3	3509.9207
P07589	Fibronectin OS = Bos taurus GN = FN1 PE = 1 SV = 4	29	2585.3821
P02543	Vimentin OS = Sus scrofa GN = VIM PE = 1 SV = 2	29	1120.5058
P04004	Vitronectin OS = Homo sapiens GN = VTN PE = 1 SV = 1	7	1645.8233
Q2KJD0	Tubulin bcta-5 chain OS=Bos taurus GN = TUBB5 PE = 2 SV = 1	5	1142.5614
P50454	Serpin HI OS = Homo sapiens GN = SERPINH! PE = 1 SV = 2	9	925.495
Q9P0S9	Trans-membrane protein 14C OS = Homo sapiens GN = TMEM14C PE = 1 SV = 1	2	2815.5473

^a^ Protein accession number; ^b^ protein description; ^c^ number of peptides matched; ^d^ expected peptide mass.

## Abbreviations

ACLF	Anterior cruciate ligament fibroblast
ADSCs	Adipose-derived stem cells
BMSCs	Bone marrow-derived mesenchymal stem cells
DAPI	4′6-Diamidino-2-phenylindole
DMEM/HG	Dulbecco’s modified Eagle medium/high glucose
DMEM/LG	Dulbecco’s modified Eagle medium/low glucose
ECM	Extracellular matrix
ELISA	Enzyme-linked immunosorbent assay
FBS	Fetal bovine serum
FGF−2	Fibroblast growth factor-2
FITC	Fluorescein isothiocyanate
FTIR	Fourier-transform infrared
GAGs	Glycosaminoglycans
IF	Immunofluorescent
MRI	Magnetic resonance imaging
MTT	3-(4,5-Dimethylthiazol-2-yl)-2,5-diphenyltetrazolium bromide
PBS	Phosphate-buffered saline
PE	Phycoerythrin
PRP	Platelet-rich plasma
SEM	Scanning electron microscopy
TBS	Tris-buffered saline
TGF-β1	Transforming growth factor beta 1
